# Cholinergic-dependent dopamine signals in mouse dorsal striatum are regulated by frontal but not sensory cortices

**DOI:** 10.1101/2025.09.30.679538

**Published:** 2025-09-30

**Authors:** Hannah C. Goldbach, Rachele Rimondini, Evan S. Swanson, Jung Hoon Shin, Michael E. Authement, Lucy G. Anderson, Han Bin Kwon, Ron Paletzki, Charles R. Gerfen, Linda M. Amarante, Richard J. Krauzlis, Veronica A. Alvarez

**Affiliations:** 1. Section on Neurobiology of Compulsive Behaviors, National Institute of Mental Health; Bethesda, MD, USA; 2. Laboratory on Neurobiology of Compulsive Behaviors, National Institute on Alcohol Abuse and Alcoholism; Rockville, MD, USA; 3. Laboratory of Sensorimotor Research, National Eye Institute; Bethesda, Maryland, USA.; 4. Center on Compulsive Behaviors, National Institutes of Health; Bethesda, Maryland, USA.; 5. Section on Neuroanatomy, National Institute of Mental Health; Bethesda, Maryland, USA

## Abstract

Everyday decisions depend on linking sensory stimuli with actions and outcomes. The striatum supports these sensorimotor associations through dopamine-dependent plasticity. Thus, the timing and magnitude of dopamine release is critical for learning. Recent work has characterized a local striatal microcircuit in which cholinergic interneurons (CINs) modulate dopamine release via acetylcholine activation of nicotinic receptors on dopamine axons. Here, we show that visual stimuli evoke dopamine responses in the dorsomedial striatum through this cholinergic-dependent mechanism. Using anatomical and functional methods to identify which pathways elicit these signals, we found that primary visual cortex and early sensory areas that project to the striatum exhibited only weak connectivity to CINs, despite robust connectivity to projection neurons, and were unable to drive dopamine release. In contrast, frontal cortical regions, including the prelimbic and anterior cingulate cortices, strongly recruited CINs and acetylcholine, producing robust dopamine release both *ex vivo* and *in vivo*. These findings reveal a fundamental distinction between sensory and frontal cortical inputs to the striatum, demonstrating that only the latter provide effective access to cholinergic-dependent dopamine signaling. This work establishes a framework for understanding how cortical circuits shape striatal dopamine to support reinforcement learning.

## Introduction

Sensory inputs, whether in the form of explicit cues or subtle hints, provide a nearly constant flux of information that guides our decisions about actions, based on expected outcomes. However, the complex pathways through which sensory information drives action remain unclear. The dorsal striatum is one brain area known to play a role in this process^1–4^. As a hub of cortical and thalamic inputs^5,6^, as well as inputs from midbrain dopamine neurons, the striatum is well-positioned to integrate contextual sensory information with expected outcomes. Historically, most work has focused on how striatal medium spiny neurons (MSNs), which comprise the main output pathways of the striatum, are driven by glutamatergic inputs and modulated by dopamine^7,8^. The temporal alignment of these signals is crucial as it determines the rules of synaptic plasticity, promoting either long-term potentiation or depression of selective inputs^9–11^ to reshape functional connectivity in the striatum and promote the associative learning of sensory cues and motor actions^12–15^.

Midbrain-originating action potentials have long been thought to have sole control over the timing of striatal dopamine signals, so much so that midbrain neuron activity is commonly interpreted as a proxy for dopamine release in the striatum. However, an alternative mechanism has recently been identified, in which acetylcholine released by cholinergic interneurons (CINs) can act locally within the striatum to regulate dopaminergic axonal excitability and dopamine release through nicotinic acetylcholine receptors (nAChRs)^16–23^, independently of soma-generated action potentials^24,25^. This local regulatory mechanism does not reflect the activity of CINs alone. Instead, CIN-evoked dopamine appears to act as a coincidence detector by requiring synchronized recruitment by the glutamatergic inputs arriving from the cortex and thalamus^19^.

Striatal CINs produce a characteristic response to novel or salient stimuli, which can strengthen as an animal learns to associate those stimuli with actions and rewards. This has been observed both in non-human primates^26–29^ and, more recently, in mice^30,31^. The recently identified ability of CINs to locally regulate dopamine opens new possibilities about the neural circuits underlying these behavioral findings. Theoretically, cortical and thalamic projections to the striatum that recruit CINs could regulate the temporal and spatial distribution of dopamine signals to shape behavior and learned associations with sensory stimuli. In support of a physiological role for this local mechanism, a recent study demonstrated that cholinergic control over dopamine in the ventral striatum contributes to effortful behavior^32^. Yet we do not know which glutamatergic inputs drive striatal CINs to provide cholinergic control over dopamine, nor how it compares across subregions of the striatum. Recent *in vivo* evidence suggests that both top-down inputs from the cortex and bottom-up inputs from the thalamus and midbrain converge to shape CIN’s multiphasic *in vivo* response to sensory cues^33^. Therefore, CINs might play an integrative role in the formation of associations for sensory-guided actions, whereby brain regions with functional connections to CINs can synchronize acetylcholine release to gain temporal and spatial control over dopamine transmission.

These previous findings raise fundamental questions about which inputs to the striatum recruit CINs to regulate dopamine transmission. It has been shown that motor cortex^20^, prelimbic cortex^22^, and some thalamic nuclei^20,34^ evoke striatal dopamine through recruitment of CINs. However, it is unknown whether sensory cortical areas, which project robustly to the striatum overall, could directly recruit CINs in the dorsal striatum. Specifically, if sensory cortices could also recruit CINs and control the timing of dopamine signals, this could allow sensory areas to regulate directly the learned associations between stimuli and rewards directly. Alternatively, if the ability to drive striatal dopamine through CINs is unique to a few areas —such as prefrontal cortex and thalamus— it would indicate a larger role for feedforward pathways through frontal cortex or those that run through sensory thalamic nuclei.

Here we identified a specific circuit involving selected cortical inputs and striatal cell-types for driving cholinergic-dependent dopamine release in the dorsomedial striatum. We show that cholinergic-dependent dopamine signals are elicited by visual stimuli, which also recruit CINs and acetylcholine release in the dorsomedial striatum. Anatomical and functional analysis revealed that prefrontal areas connect far more heavily with CINs than primary sensory areas, despite the presence of prominent projections to striatal medium spiny neurons from primary visual cortex and other sensory cortices. We then considered what these results might mean for cholinergic control over local dopamine signaling. Fast-scan cyclic voltammetry demonstrated that primary sensory areas cannot drive cholinergic-dependent dopamine signals, while frontal cortical areas can. Lastly, *in vivo* fiber photometry confirmed these results in intact animals, demonstrating that stimulation of frontal cortical inputs drive CINs, acetylcholine release, and dopamine release in the dorsomedial striatum. Through this combination of experimental approaches, our findings establish that frontal cortices, but not sensory cortices, can play a prominent role in controlling striatal CINs and modulating striatal dopamine. The selectivity of this mechanism carries significance: only cortical areas with cognitive associative functions are capable of this local control over striatal dopamine, perhaps pointing to an evolutionary relevance of limiting which areas exert this control.

Our findings support the view that frontal areas, such as anterior cingulate area (ACA) and prelimbic cortex (PL), can contribute to stimulus-evoked dopamine signals via their inputs to striatal CINs. These results complement other findings highlighting the importance of frontal cortices in sensorimotor learning and dopamine transmission, and suggest that the details of these circuits may prove critical for understanding the etiology of dopamine-related disorders.

## Results

### Salient sensory stimuli drive striatal CINs, acetylcholine, and dopamine

**Figure 1:**
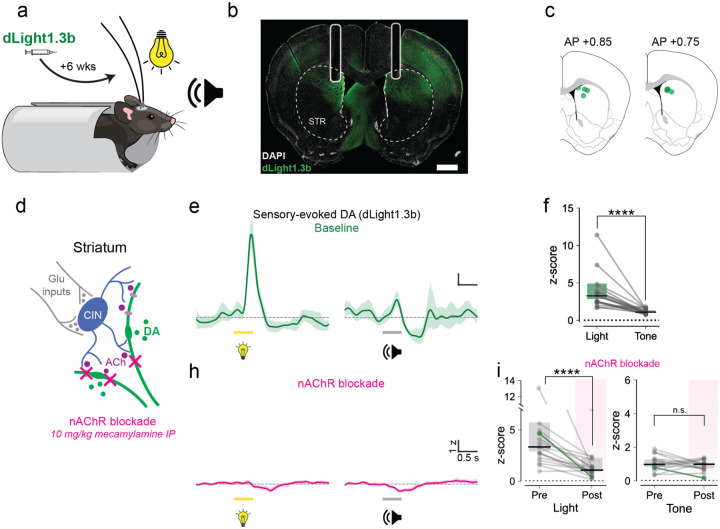
Salient sensory stimuli trigger striatal dopamine **a**. Experimental setup. Mice expressing dLight and implanted in the dorsomedial striatum were head-fixed and presented with white light (500 ms, 5.9 cd/m^2^) and tones (7.5 kHz, 500 ms), spaced roughly every 30 seconds. **b-c**. Representative image of dLight expression from one mouse and fiber placements in DMS. **d**. Diagram of striatal microcircuitry under study: cholinergic interneurons (CIN, blue) are driven by glutamatergic inputs (gray), leading to release of acetylcholine (purple), which activates nAChRs on dopamine neuron axons (green) to produce local dopamine release. **e**. Single session example traces of dopamine responses aligned to light (left) and tone (right) onset. **f**. Maximum z-score dopamine responses to light and tone (n = 18 hemispheres from 14 mice). **g**. The nAChR antagonist mecamylamine (10 mg/kg, i.p.) prevented stimulus-evoked dopamine response. **h**. Across-animal effect of mecamylamine on light-evoked (left) and tone-evoked (right) dopamine responses (light: p < 0.0001; tone: p = 0.52, Wilcoxon Signed Rank test). For all traces, line and shade represent mean z-score +/− bootstrapped 95% CI. For all bar plots, symbols represent data from one hemisphere. Black lines represent medians, and gray bars represent the bootstrap 95% CI.

We first used fiber photometry and dLight, a fluorescent dopamine sensor, to survey dopamine release in response to salient sensory stimuli (Fig. 1a–d, Methods). Mice expressing dLight1.3b were implanted with a headframe and fiber optic cannula in the dorsomedial striatum (DMS). Through these cannulas, we recorded fluorescent signals as mice passively experienced alternating visual stimuli (LED light, 5.9 cd/m^2^ as measured on the floor of the box) or auditory tones (7.5kHz), each lasting 500ms and occurring roughly 30s apart. Dopamine responses to the visual stimulus were three times as large as those to the auditory stimulus (Fig. 1e–f; light: median 3.27 [2.81 – 4.92 bootstrap 95% CI] z-score vs tone: median 1.08 [1.02 – 1.31], n = 18 hemispheres from 14 mice; p < 0.0001, Wilcoxon Signed Rank test).

Recent papers in rodents have focused on the interactions between dopamine and acetylcholine in the dorsal striatum^23,32,35–37^. In some contexts, acetylcholine can amplify striatal dopamine, while in other contexts, acetylcholine may suppress dopamine signals. To explore whether some component of these sensory-evoked dopamine responses is dependent on acetylcholine, we systemically administered saline (Fig. S1) or mecamylamine, a non-competitive nicotinic receptor antagonist (Fig. 1g), while mice experienced the same stimuli. Mecamylamine significantly decreased visually-evoked dopamine responses (Fig. 1h–i; n = 18 hemispheres from 14 animals, p < 0.0001, Wilcoxon Signed Rank). However, it had little effect on tone-evoked dopamine responses (Fig. 1h–i; n = 15 hemispheres from 11 animals; p = 0.52, Wilcoxon Signed Rank).

**Figure 2:**
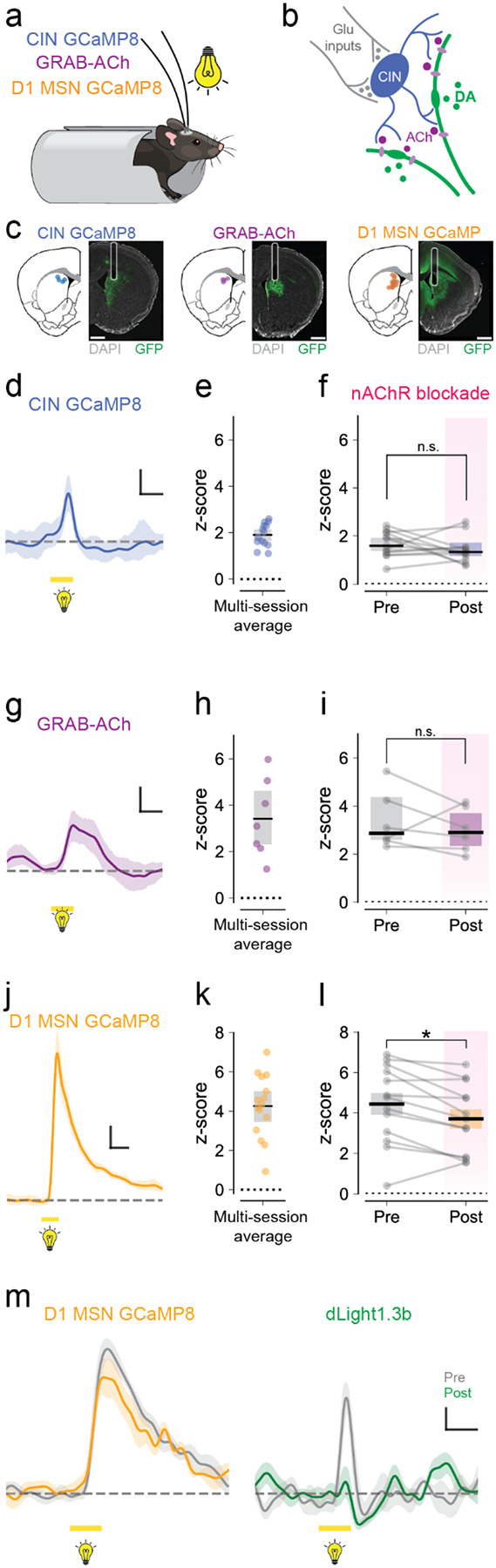
Visual stimuli drive striatal CINs and acetylcholine responses **a**. Experimental setup. Mice expressing GCaMP in CINs, or D1-MSNs, or GRAB-ACh in the dorsomedial striatum were head-fixed and presented with sensory stimuli: light (5.9 cd/m^2^, 500 ms) and tones (7.5 kHz, 500 ms), interleaved every 30s. **b**. Diagram of the striatal microcircuitry possibly involved: cholinergic interneurons (blue) are driven by glutamatergic striatal inputs (gray) and release acetylcholine (purple), which binds to nAChRs on DA axons (green) to produce local dopamine release **c**. Fiber placements and histology examples from mice expressing GCaMP8 in CINs, GRAB-ACh, or GCaMP8 in D1 MSNs. Scale bars are 1 mm. **d, g, j**. Single session example traces of GCaMP responses in CINs (d), GRAB-ACh (g), and GCaMP responses in D1-MSNs (j) aligned to light onset. Lines and shades are mean z-score +/− bootstrapped 95% CI. Scale bars are 0.5 z-scored fluorescence on the y-axis and 0.5 s on the x-axis. **e, h, k**. Maximum z-score of light-evoked responses for GCaMP in CINs (e), GRAB-ACh (h), and GCaMP in D1-MSNs (k). Symbols are data from one hemisphere. Black lines represent means, and gray bars represent bootstrapped 95% CI. **f, i, l**. Effect of nAChR antagonist mecamylamine (10 mg/kg, i.p.) on in vivo responses to light onset in mice expressing GCaMP in CINs (f, p = 0.45, Wilcoxon Signed Rank), GRAB-ACh (i, p = 0.44) or GCaMP in D1-MSNs (k, p = 0.01). **m**. Representative traces from one animal expressing GCaMP8 in D1-MSNs in one hemisphere (left) and dLight1.3b in the other hemisphere (right). GCaMP in D1-MSN was only slightly reduced, while dLight1.3b was dramatically reduced by mecamylamine administration. For all traces, line and shade represent mean z-score +/− bootstrapped 95% CI. For group plots, gray symbols and lines are paired data from a single hemisphere pre- and post- (pink) mecamylamine administration. Black lines and shaded bars represent the median and bootstrapped 95% CI.

Striatal CINs have been shown to respond to salient sensory stimuli in a series of classical electrophysiology studies done in non-human primates^26,27^. To assess whether the visual stimulus recruited striatal CINs and evoked local ACh release in DMS, we expressed unilateral GCaMP8 in CINs and/or unilateral GRAB-ACh3.0, an acetylcholine sensor (Fig. 2a–c, Table S1). Calcium signals from CINs increased in response to visual stimuli (Fig. 2d–e; median 1.92, [1.65 – 2.15] z-score, n = 14 hemispheres from 12 animals). These responses were not affected by mecamylamine (Fig. 2f; n = 13 hemispheres from 11 animals; p = 0.45, Wilcoxon Signed Rank). Similarly, visual stimuli produced increases in acetylcholine, as measured with GRAB-ACh3.0 (Fig. 2g–h; median 3.42 [2.31 – 4.62] z-score, n = 7 hemispheres from 7 animals). These visually-evoked acetylcholine responses were unaffected by mecamylamine (Fig. 2i; n = 6 hemispheres from 6 animals; p = 0.44, Wilcoxon Signed Rank).

Systemic administration of mecamylamine might affect other pathways and impair the ability of sensory information to reach the striatum. To control for this, we repeated these experiments in a cohort of mice expressing GCaMP8s in D1 MSNs, which responded robustly to the visual stimulus (Fig. 2j–k; median 4.77 [3.41 – 5.42] z-score, n = 13 hemispheres from 8 animals). Calcium signals in D1 MSNs prevailed after mecamylamine, but showed a small decrease (Fig. 2l–m; n = 13 hemispheres from 8 animals; p = 0.01, Wilcoxon Signed Rank), possibly due to the blunting of the dopamine response by mecamylamine. The lasting D1, CIN, and acetylcholine responses in the presence of mecamylamine strongly suggested that visual information was still reaching the striatum and argued against impairment of the pathways that carry visual information to the striatum. Meanwhile, the dramatic decrease in dopamine response by mecamylamine (Fig. 2m) indicates that acetylcholine and nicotinic ACh receptors contribute to the sensory stimuli-evoked dopamine response.

### Unlike frontal cortical areas, primary sensory areas do not synapse onto dorsal striatal CINs

**Figure 3.**
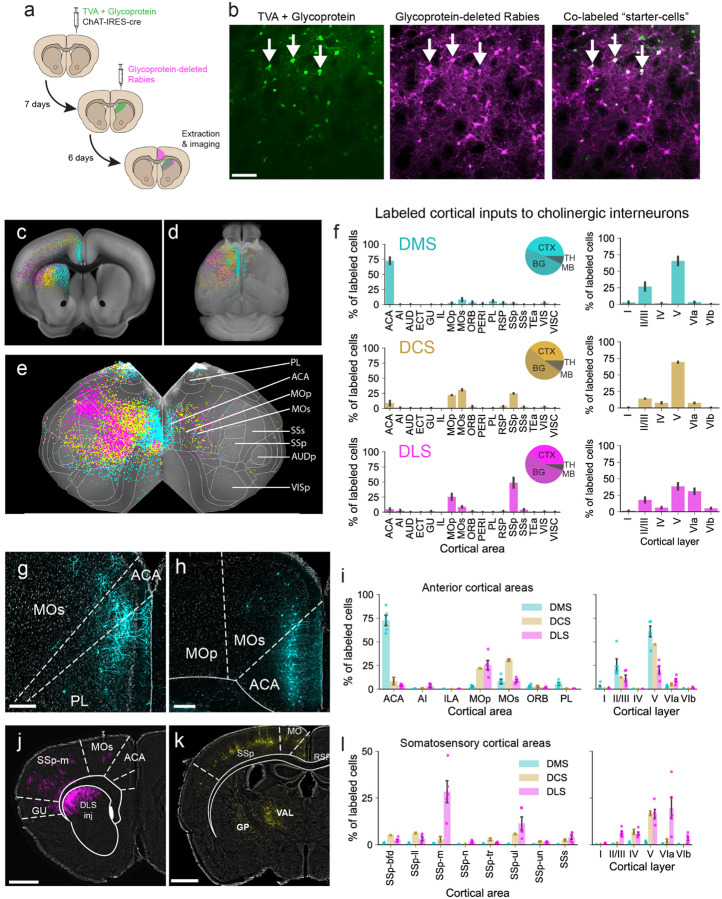
Retrograde rabies tracing demonstrates a widespread anterior bias in cortical connectivity to striatal cholinergic interneurons **a**. Experimental timeline for injecting ChAT-IRES-cre mice with cre-dependent TVA rabies receptor + glycoprotein, followed by a modified glycoprotein-deleted retrograde rabies vector to label CIN projecting cells. **b**. Images of a DMS injection area showing cells expressing TVA-glycoprotein (left, green) and glycoprotein-deleted rabies (middle, magenta). Overlaid images (right) show co-labeled cells that serve as “starter” cells (white). **c, d, e**. Reconstructions showing CIN-projecting cortical cells in coronal view (c), whole-brain view (d), and flattened brain view (e) from injections in DMS (cyan), DCS (yellow), and DLS (magenta). **f**. Quantifications of CIN-projecting labeled cortical cells per area (left) and per cortical layer (right). Plots show averages of all labeled cortical cells for DMS, DCS, and DLS injection sites. ACA, anterior cingulate area; PL, prelimbic; MOp, primary motor; MOs, secondary motor; SSp, primary somatosensory. See Table S2 for other anatomical nomenclature. Inset pie charts represent percentages of labeled cells in cortex (CTX), basal ganglia (BG), thalamus (TH), and midbrain (MB). **g-h, j-k**. Representative images of retrogradely labeled cortical neurons projecting to striatal CINs in DMS (g-h), to CINs in DCS (j), and to CINs in DLS (k) in coronal sections. Inset scale bars are 250 μm. **i, l**. Quantifications of retrogradely labeled cortical cells per subregion (left) and layer (right) expressed as a percentage of total labeled cortical cells for frontal cortical areas (i) and somatosensory areas (l).

Next, we asked which striatal inputs could drive striatal CINs to promote local acetylcholine and, potentially, dopamine release. Primary sensory areas (visual, somatosensory, and auditory cortex) are known to project to the dorsal striatum, where they form synapses with MSNs. Therefore, it could be possible that they could also synapse onto CINs to drive responses to salient sensory stimuli. To address this, we used cell-type-specific monosynaptic retrograde rabies tracing to widely survey the frequency of cortical connections to striatal CINs in the dorsal striatum. We virally expressed cre-dependent TVA receptors and rabies glycoprotein in striatal CINs of ChAT-cre mice, which allowed a modified glycoprotein-deleted rabies virus to selectively infect cre-expressing CINs (“starter cells”) and label neurons that synapse onto them (Fig. 3a–b; Table S1; Fig. S2; Methods). The majority of labeled projection cells were identified in the cortex (49–54%), though labeled cells also included those projecting from the thalamus (5–9%), midbrain (1–2%), and locally within basal ganglia (38–44%; Fig. 3f, **insets**)

Labeling across the medial to lateral axis in the cortex mirrored injection sites across the same axis in the striatum. Injections in medial, central, and lateral dorsal striatum produced labeling in medial frontal areas, motor cortices, and somatosensory cortex, respectively (Fig. 3c–f). Most of this labeling was found in anterior cortical areas, with only sparsely labeled cells in more posterior areas, including primary visual cortex (VISp) and primary auditory cortex (AUDp, Fig. 3c–f).

Injections in the DMS led to robust labeling in the anterior cingulate area (ACA) with only sparsely labeled cells in sensory cortices (Fig. 3f, left). The majority of cells labeled in the cortex were located in layer 5 (66% of labeled cortical cells), though a smaller percentage were located in layers 2/3 (27%) (Fig. 3f, right), as expected for striatal projecting neurons^5,6,38–41^. Of all cortical cells labeled per mouse, an average of 73% were located in ACA (Fig. 3g–i), with additional labeling in the secondary motor cortex (8% of cortical cells, MOs) and prelimbic cortex (6%, PL) (Fig. 3g, i). Very few labeled cells were located in primary somatosensory cortex (SSp, 2%), VISp (3%), or AUDp (0.4%) (Fig. 3f, Fig. S2). Injections in the dorsal central striatum (DCS) led to fairly even labeling across primary motor cortex (MOp, 22% of cortical cells), secondary motor cortex (MOs, 31%), and SSp (25%). Meanwhile, injections in the dorsal lateral striatum (DLS) led to labeling in the heaviest expression in SSp (49% of labeled cortical cells) and lesser expression in MOp (25%) and MOs (9%, Fig. 3f,k). Again, the majority of these cells in both DCS and DLS were located in layer 5 (69% and 38%, respectively), with smaller percentages in layer 6 (8%, and 31%) and layers 2/3 (14% and 18%) (Fig. 3f).

SSp can be divided into seven topographic sub-regions, each representing a part of the body. When we broke down the DLS cell counts further into these sub-areas, we found that roughly half of the labeled SSp cells were located in the mouth/jaw area (SSp-m, 28% out of a total 49%), and one quarter of SSp cells were in the upper limb (SSp-ul, 11% out of 49%, Fig. 3k–l). Barrel field of somatosensory cortex (SSp-bfd) contained only 2% of the labeled cells across the cortex (Fig. 3k–l). In comparison, 3% of all labeled cells were also found across the visual cortex, which can also be broken down into several sub-regions. VISp contained only 0.6% of labeled cells, while secondary visual areas (VISa, VISal, VISam, VISl, VISli, VISpm, and VISrl) contained less than 1% (0.05 – 0.7%) of labeled cells (Fig. S2). Auditory cortex contained even fewer labeled cells than the visual cortex, with only 0.03 to 0.2% of cells in each auditory sub-region (Fig. S2).

These results demonstrate that only a handful of medial frontal cortical areas synapse frequently with striatal CINs in the dorsomedial striatum.

### Frontal areas form reliable functional connections with striatal CINs, while primary sensory areas do not

**Figure 4:**
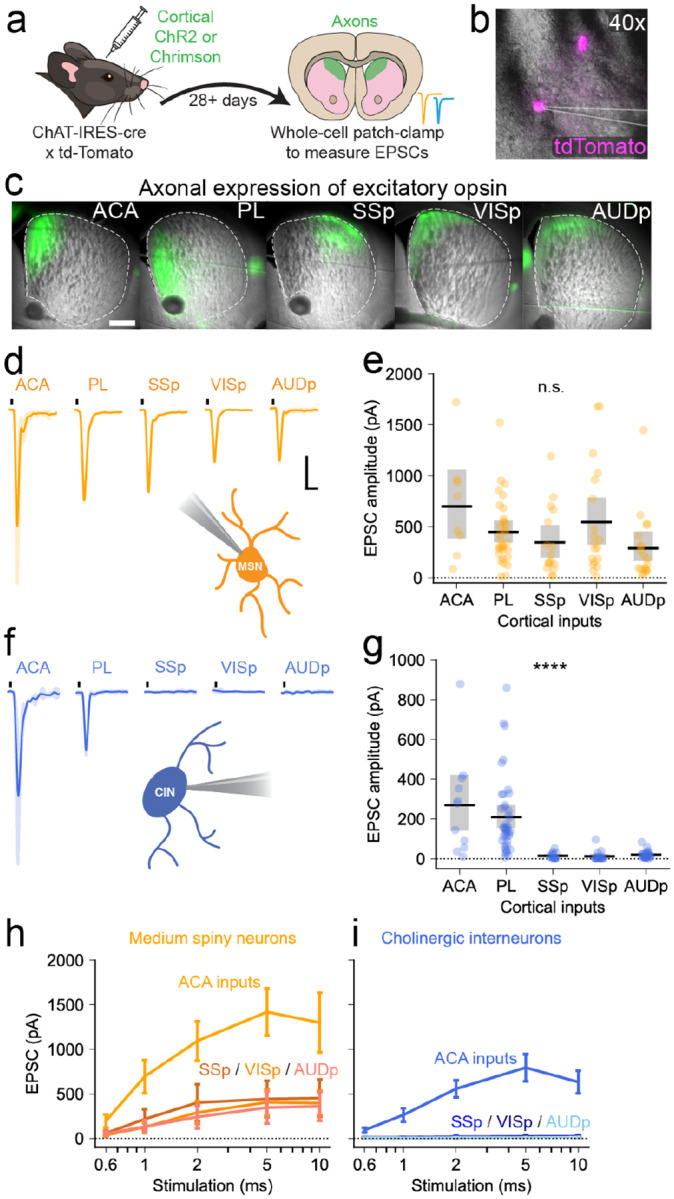
Frontal cortical areas form reliable functional connections with striatal CINs, while sensory cortical areas do not **a**. Mice with labeled CINs (ChAT-IRES-Cre x tdTomato) were injected in frontal or sensory cortical areas with either ChR2-eYFP or ChrimsonR-tdTomato. Brains were extracted and sliced for ex vivo whole-cell patch clamp electrophysiology following a minimum four-week incubation. **b**. Visualization of genetically encoded tdTomato expression in striatal CINs, as viewed on the electrophysiology rig. **c**. Axonal expression of virally-encoded excitatory opsins, with injections either in anterior cingulate (ACA), prelimbic (PL), somatosensory (SSp), visual (VISp), or auditory (AUDp) cortices. Scale bar is 500 μm. **d**. Representative EPSC traces recorded from MSNs in response to optogenetic stimulation of cortical areas. Scale bar is 100 pA on the y-axis and 100 ms on the x-axis. **e**. Average MSN EPSC amplitudes evoked via optogenetic stimulation of cortical areas (means across areas not significantly different, p = 0.06, Kruskal-Wallis test). **f**. Representative EPSC traces recorded from CINs in response to optogenetic stimulation of cortical areas. **g**. Average CIN EPSC amplitudes evoked via optogenetic stimulation of cortical areas (means across areas are significantly different, p < 0.0001, Kruskal-Wallis test). h. Input-output curves showing EPSC amplitudes recorded from MSNs (orange) and CINs (blue) in response to a range of stimulation durations. All traces are mean and bootstrap 95% CI. For group plots, symbols represent data from individual cells; black lines and shaded bars represent median and bootstrap 95% CI.

The retrograde rabies tracing helped identify a few cortical areas that contain many cells synapsing onto striatal CINs, but it does not inform us about the strength of those synapses. In a divergent connectivity pattern, a small number of cortical neurons may form many, strong synapses with CINs, while in a convergent pattern, a large number of cortical neurons may form few weak synapses with CINs. We therefore sought to validate functionally the rabies results using *ex vivo* whole-cell patch-clamp electrophysiology to record excitatory postsynaptic currents (EPSCs) from striatal MSNs and CINs as we optogenetically stimulated different cortical inputs. ChAT-tdTomato mice with fluorescently labeled CINs were used to aid in cell identification. These mice were injected with ChR2-eYFP or ChrimsonR-tdTomato in either the anterior cingulate cortex, prelimbic cortex, primary somatosensory, visual, or auditory cortex (Methods; Fig. 4a–c).

Coronal brain slices showed distinctive patterns of labeled corticostriatal projections in the dorsomedial or dorsolateral striatum, depending on the targeted cortical areas (Fig. 4c). CIN and MSN recordings were made from the striatal region receiving labeled projections. 405 nm blue light (1.3 mW) and 590 nm orange light (0.7 mW) were used to optogenetically excite these terminals, as a subset of these animals expressed both opsins (see below, Fig. S3). When recording from MSNs, optogenetic activation (1 ms) of ACA or PL inputs produced large excitatory postsynaptic currents (Fig. 4d–e, ACA: mean 699.4 [384.9 – 1056.7 bootstrap 95% CI] pA, n = 8 cells from 3 animals; PL: 448.1 [344.9 – 560.7] pA, n = 32 cells from 9 animals). In line with previous findings, sensory areas also produced measurable EPSCs in MSNs (Fig. 4d–e, SSp: mean 346.2 [199.4 – 518.2 bootstrap 95% CI] pA, n = 16 cells from 5 animals; VISp, 544.0 [327.2 – 792.4] pA, n = 19 cells from 5 animals; AUDp, 289.1 [165.4 – 452.6] pA, n = 19 cells from 5 animals). The tested cortical regions all formed robust synaptic connections with MSNs and no significant difference in the mean responses was found (p = 0.06, Kruskal-Wallis test).

When recording from CINs, however, we only saw large EPSCs in response to a 10 ms stimulation of ACA or PL (Fig. 4f–g, ACA: 269.6 [143.6 – 423.3] pA, n = 11 from 3 animals; PL: 209.4 [155.2 – 270.6] pA, n = 41 cells from 12 animals). Stimulation of inputs from primary sensory areas (10 ms) consistently produced little to no response in CINs (Fig. 4f–g, SSp: 14.7 [7.3 – 23.2] pA, n = 14 cells from 4 animals; VISp: 12.2 [3.8 – 24.2] pA, n = 18 cells from 7 animals; AUDp: 20.2 [13.8 – 28.0] pA, n = 25 cells from 6 animals). Comparing the strength of the synaptic connections to CINs across the cortical regions revealed significant differences between ACA/Pl and the other sensory areas (p < 0.0001, Kruskal-Wallis test). We explored the responses in CIN and MSNs with a range of stimulation parameters, namely the duration of the stimulation (Fig. 4h–i). When considering the sensory areas, a 2–5 ms stimulation was sufficient to saturate the responses recorded from MSNs (Fig. 4h). Meanwhile, a 10 ms stimulation of sensory terminals failed to produce any response in CINs (Fig. 4i).

As an additional test of the selective connectivity from sensory cortex to MSNs and not CINs, a cohort of mice simultaneously expressed Chrimson in a primary sensory area and ChR2 in the frontal cortex (ACA / PL), which served as an internal control. This allowed us to compare how the same cell (a single MSN or single CIN) responded to two different cortical inputs (Fig. S3). The paired data confirmed the findings that single CINs responded strongly to inputs from PL, but not any primary sensory area. This data also confirmed that single MSNs receive inputs from both frontal and sensory cortices. This experiment provided the benefit of paired data and internal control, but also restricted our recordings spatially to places with overlapping expression.

Our rabies tracing results unexpectedly showed labeling in the mouth region of the somatosensory cortex (SSp-mj). When we recorded from MSNs in response to stimulation of SSp-m terminals, we observed measurable EPSCs (Fig. S4; 335.0 [112.5 – 616.2] pA; n = 9 cells from 3 animals; mean [bootstrap 95% CI]). When we recorded from CINs in response to the same terminals, we observed smaller, more infrequent EPSCs (mean [bootstrap 95% CI], 82.8 [25.4 – 156.3] pA; n = 8 cells from 3 animals; mean [bootstrap 95% CI]). These findings are consistent with those we found when stimulating other somatosensory areas, such as SSp-bfd terminals.

In summary, the *ex vivo* electrophysiology data functionally confirmed the anatomical findings from Figure 3. While MSNs received inputs from all areas tested, we observed a heavy bias in connectivity towards frontal areas when recording from CINs.

### Frontal cortical areas, but not sensory, produce local acetylcholine-dependent dopamine release

**Figure 5.**
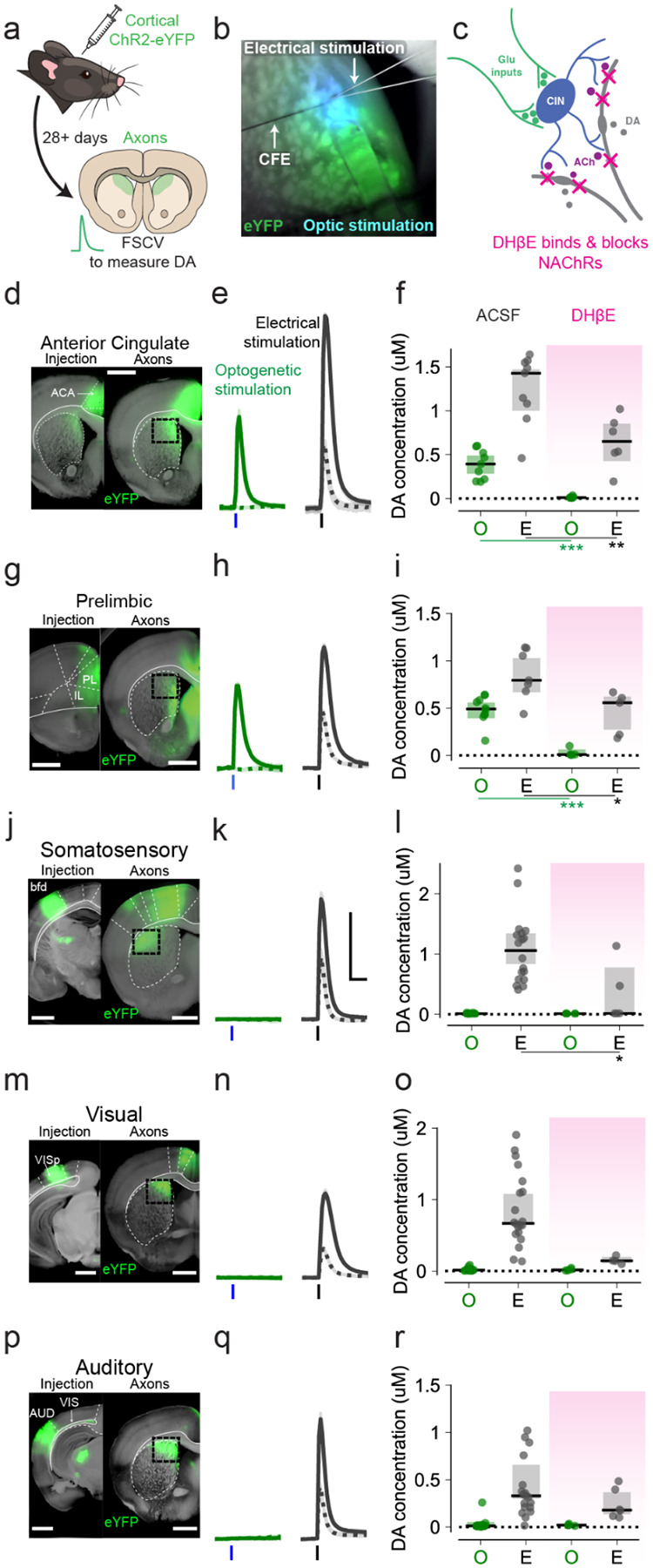
Frontal areas, but not sensory areas, synchronously activate striatal CINs to produce local dopamine release ex vivo **a**. Mice were injected with ChR2-eYFP in frontal or sensory cortical areas. Following a minimum four-week incubation, brains were extracted and sliced for ex vivo fast-scan cyclic voltammetry to measure electrically and optogenetically evoked dopamine. **b**. Example slice prep for fast-scan cyclic voltammetry, using a carbon fiber electrode (CFE) to measure dopamine evoked through either electrical stimulation (glass pipette) or optogenetic stimulation (optic fiber, blue light) of ChR2-eYFP expression (green). **c**. Diagram of the striatal microcircuitry involved: cholinergic interneurons (blue) are driven by glutamatergic striatal inputs (gray) and release acetylcholine (purple), which binds to nAChRs on DA axons (green) to produce local dopamine release. DHβE application blocks nAChRs to prevent DA release. **d, g, j, m, p**. Left, ChR2-eYFP expression (green) in the injection area: anterior cingulate cortex (d), prelimbic cortex (g), barrel fields of somatosensory cortex (SSp-bfd, j), visual cortex (VIS, m), auditory cortex (AUD, p). Right, ChR2-eYFP expressing projections (green) from the respective cortical areas in dorsomedial (d, g, m, p) and dorsolateral (j) striatum. The black box shows the site of dopamine recordings. All white scale bars are 1 mm. **e, h, k, n, q**. Single-slice average of dopamine transients, as measured with fast-scanning cyclic voltammetry (FSCV). Gray traces show dopamine transients elicited by electrical stimulation. Green traces show the responses elicited by optogenetic stimulation of ChR2 at the same recording site. Dashed gray and green lines show the remaining dopamine responses after washing in a nicotinic receptor blocker (1 μM DHβE). Blocking nicotinic receptors abolished optogenetically evoked transients and greatly reduced electrically evoked transients in all cases (shaded error bars are ± SEM). Scale bars are 0.5 μM on the y-axis and 0.5 s on the x-axis. **f, i, l, o, r**. Average dopamine transient amplitudes elicited by electrical stimulation (gray bars) and optogenetic stimulation (green bars) for animals expressing ChR2-eYFP in ACA (f), PL (i), SSp-bfd (l), VIS (o), and AUDp (r) cortical regions. Each dot represents data from one slice. Pink-shaded data were collected after DHβE bath application. (error bars are median ± bootstrap 95% CI; p < 0.05*; p < 0.01**; p < 0.001***; non-significant pairs are not shown, Mann-Whitney U test).

Our *in vivo* results in Figure 1 showed that the visual stimulus-evoked dopamine responses might partially be contingent on acetylcholine. Therefore, we next sought to determine whether the cortical inputs identified in Figure 2 and tested functionally in Figure 3 were capable of driving striatal dopamine release via an acetylcholine-dependent mechanism. We expressed an excitatory opsin (either ChR2 or Chrimson) in various cortical areas of C57B6/J mice (Methods; Fig. 5a) and used *ex vivo* fast scanning-cyclic voltammetry in *ex vivo* brain slices to measure dopamine transients elicited by optogenetic stimulations (470 nm, 5 ms, 1.3 mW; Fig. 5b–c) and short electrical pulses that serve as a positive control (0.2 ms, 400 uA). Optogenetically stimulating ACA terminals consistently produced dopamine transients in DMS, although electrically evoked dopamine transients in this area were slightly larger, likely due to the additional signal from direct stimulation of dopamine axons (Fig. 5d–f; optogenetic: median 0.39 [0.29 – 0.49 bootstrap 95% CI] μM, n = 9 slices from 3 animals; electrical: 1.43 [1.00 – 1.47] μM, n = 9 slices from 3 animals). Stimulating PL corticostriatal terminals produced similar results (Fig. 5g–i; optogenetic: 0.49 [0.39 – 0.56] μM, n = 10 slices from 4 animals; electrical: 0.79 [0.67 – 1.03] μM, n = 7 slices from 3 animals). To confirm that the cortically-evoked dopamine signal in the slice is a local signal dependent on CIN recruitment and acetylcholine acting on nAChRs, we washed on a high concentration of DHβE (1 μM), a nicotinic acetylcholine receptor antagonist, which completely abolished any optogenetically evoked dopamine transients and significantly reduced electrically evoked events (Fig. 5f; ACA optogenetic DHβE: 0.011 [0.0096 – 0.025] μM, n = 6 slices from 3 animals; ACA electrical DHβE: 0.65 [0.42 – 0.86] μM, n = 6 slices from 3 animals; ACA optogenetic ACSF vs DHβE, p = 0.001; electrical ACSF vs DHβE, p = 0.007, Mann Whitney U test; Fig. 5i; PL optogenetic DHβE: 0.026 [0.0064 – 0.063] μM, n = 5 slices from 3 animals; PL electrical DHβE: 0.45 [0.27 – 0.62] μM, n = 5 slices from 3 animals; PL optogenetic ACSF vs DHβE, p = 0.0007; PL electrical ACSF vs DHβE, p = 0.02, Mann Whitney U test). In contrast, we found that optogenetically stimulating sensory (SSp, VISp, or AUDp) corticostriatal terminals failed to produce any measurable dopamine transients in either DMS or DLS (Fig. 5j–l, SSp: 0.011 [0.0089 – 0.014] μM, n = 18 slices from 5 animals; Fig. 5m–o, VISp: 0.015 [0.013 – 0.30] μM, n = 19 slices from 8 animals; Fig. 5q–r, AUDp: 0.011 [0.0098 – 0.054] μM, n = 19 slices from 4 animals). Interleaved electrical stimulation consistently produced dopamine transients in the same brain slices and under the same conditions (Fig. 5k–l, SSp: 1.06 [0.83 – 1.34] μM, n = 18 slices from 5 animals; Fig. 5n–o, VISp: 0.67 [0.65 – 1.08] μM, n = 20 slices from 8 animals; Fig. 5q–r, AUDp: 0.33 [0.30 – 0.67] μM, n = 19 slices from 4 animals).

We also tested SSp-m to determine whether the connectivity seen with retrograde rabies tracing (Fig. 3) was strong enough to produce dopamine. Consistent with other sensory areas, this specific region of somatosensory cortex was also unable to evoke dopamine despite strong electrically-evoked transients in the same recorded region (Fig. S4; optogenetic: 0.015 [0.013 – 0.022] μM, n = 10 slices from 3 animals; electrical: 0.92 [0.84 – 1.40] μM, n = 10 slices from 3 animals).

Together, these experiments provide *ex vivo* evidence that, unlike sensory cortices, frontal cortical areas such as ACA and PL produce dopamine release through a local mechanism requiring nAChRs, and further suggest that these select corticostriatal inputs can drive synchronized activation of cholinergic interneurons.

### Frontal areas can drive striatal dopamine, CINs, and acetylcholine release *in vivo*

We last sought to test whether in vivo optogenetic stimulation of PL/ACA terminals in DMS can evoke dopamine signals through the local recruitment of CINs and subsequent ACh release. Mice (wildtype or ChAT-Cre) were injected bilaterally in PL/ACA with ChrimsonR-tdTomato and in the DMS with either a dopamine sensor (dLight1.3b), cre-dependent calcium sensor (DIO-GCaMP8), or acetylcholine sensor (GRAB-ACh) in separate hemispheres. These mice were implanted bilaterally with fiber-optic cannulae, through which we could simultaneously record fluorescent changes and stimulate cortical terminals in DMS with short pulses of red light (625 nm, 5.5 mW; single pulse or 3 and 10 pulses at 20 Hz, 30s interval).

**Figure 6:**
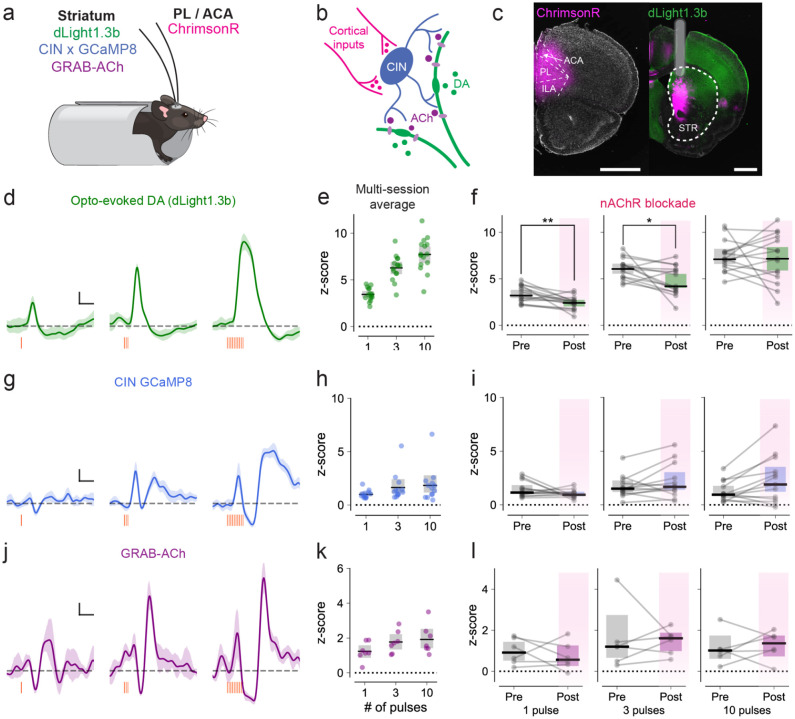
Stimulation of frontal cortical terminals triggers striatal dopamine, CINs, and acetylcholine release **a**. Experimental setup. Mice expressing Chrimson-tdTomato in prelimbic or anterior cingulate cortex and dLight, GCaMP8 in CINs, or GRAB-ACh in the dorsomedial striatum were implanted and head-fixed for optogenetic stimulation of frontal cortical terminals in the striatum. **b**. Diagram of the striatal microcircuitry potentially involved: cholinergic interneurons (blue) are driven by ChrimsonR-expressing glutamatergic striatal inputs (magenta) and release acetylcholine (purple), which binds to nAChRs on DA axons (green) to produce local dopamine release. **c**. Example ChrimsonR-tdTomato expression in frontal cortical areas (pink) and dLight1.3b expression in striatum. **d, g, j**. Representative dLight1.3b (d), CIN GCaMP (g), and GRAB-ACh (j) responses to single 10 ms pulses of 625 nm red light (left) and trains of three pulses at 20 Hz (center) or trains of 10 pulses at 20 Hz (right). **e, h, k**. Maximum z-scored responses evoked from each hemisphere expressing dLight1.3b (e), CIN GCaMP (h), and GRAB-ACh (k). **f**. nAChR blockade with 10 mg/kg mecamylamine i.p. reduced responses evoked by single pulses of light (p = 0.005, Wilcoxon Signed Rank test) or 3-pulse trains (p = 0.01, Wilcoxon Signed Rank test). nAChR blockade did not affect the responses evoked by a 10 pulse train (p = 0.85, Wilcoxon Signed Rank test). **i**. nAChR blockade did not affect CIN GCaMP responses (1 pulse: p = 0.64, 3 pulses: p = 0.11, 10 pulses: 0.07, Wilcoxon Signed Rank test). **l**. nAChR blockade did not affect GRAB-ACh responses (1 pulse: p = 0.56, 3 pulses: p = 0.84, 10 pulses: 0.84, Wilcoxon Signed Rank test).

Even a single 10 ms pulse of red light evoked measurable striatal dopamine responses (Fig. 6d–e, mean 3.68 [3.26 – 4.11 bootstrap 95% CI] z-score, n = 15 hemispheres from 11 animals, averaged across sessions). The amplitude of the dopamine response increased as the number of pulses increased, with signs of saturation and altered dynamics with the longest stimulation train (Fig. 6d–e, 3 pulses: 6.45 [5.89 – 6.96] z-score; 10 pulses: 7.94 [7.29 – 8.60] z-score, averaged across sessions). To test whether a component of this evoked response was dependent on acetylcholine, we injected 10 mg/kg mecamylamine intraperitoneally to block nAChRs. Blocking nAChRs produced a significant decrease in dopamine evoked by a single pulse and a 3-pulse train (Fig. 6f; single pulse, pre-injection: median 3.20 [2.93 – 3.81 bootstrap 95% CI] z-score; single post-injection: 2.42 [2.04 – 2.74]; p = 0.005, Wilcoxon Signed Rank; 3-pulse train, pre-injection: median 6.06 [5.54 – 6.62], post-injection: median 4.20 [4.0 – 5.52]; p = 0.01, Wilcoxon Signed Rank; n = 15 hemispheres from 11 animals). These decreases with a single pulse and short trains of stimulation reveal an acetylcholine-dependent contribution of the dopamine signals. Mecamylamine had minimal effect on the *in vivo* responses evoked by more prolonged stimulation (Fig. 6f; 10 pulses, pre-injection: 7.10 [6.6–8.2], post-injection: 7.16 [5.87 – 8.40]; p = 0.85, Wilcoxon Signed Rank), resembling what has been shown *ex vivo*^42,43^.

To determine whether these cortical inputs recruit striatal CINs and produce acetylcholine signals *in vivo*, we recorded from mice that expressed GCaMP8 in CINs and/or GRAB-ACh. We observed clear responses from both sensors after stimulation of PL/ACA projections, which were biphasic and consistent with the characteristic striatal CIN burst-pause-burst pattern (Fig. 6g,j). For the quantitative analysis, we selected a time window corresponding to the first burst. A single pulse of PL/ACA terminal stimulation produced both CIN calcium responses (Fig. 6g–h; median 1.0 [0.83 – 1.21 bootstrap 95% CI] z-score, n = 13 hemispheres from 11 animals) and acetylcholine responses (Fig. 6j–k; median 1.23 [0.87 −1.59] z-score, n = 7 hemispheres from 7 animals). The response of both sensors scaled with increasing stimulation (CIN GCaMP, 3 pulses: 1.64 [1.12 – 2.41] z-score; 10 pulses: 1.84 [1.18 – 2.75] z-score; GRAB-ACh, 3 pulses: 1.77 [1.34 – 2.22] z-score; 10 pulses: 1.92 [1.40 – 2.53] z-score), suggesting larger CIN recruitment and acetylcholine release in response to prolonged stimulation of frontal cortical inputs to DMS. As hypothesized, neither sensor decreased significantly when challenged with an nAChR blockade (CIN GCaMP, 1 pulse: p = 0.64; 3 pulses: p = 0.11; 10 pulses: p = 0.07; GRAB-ACh, 1 pulse: p = 0.56; 3 pulses: p = 0.84; 10 pulses: p = 0.84; Wilcoxon Signed Rank test). Saline administration did not produce a significant change, regardless of stimulation or sensor (Fig. S5).

Altogether, these in vivo experiments offer compelling evidence that brief activation of PL/ACA projections to the dorsomedial striatum can evoke cholinergic-dependent dopamine signals, via the local recruitment of CINs and subsequent ACh release.

## Discussion

This study provides new insights into how behaviorally relevant stimuli control dopamine transmission in the striatum. Using a combination of anatomical and functional experiments, both *in vivo* and *ex vivo*, we identified which cortical circuitry can modulate striatal dopamine, and which cannot. We show that visual stimuli elicit striatal dopamine through a cholinergic-dependent mechanism involving recruitment of CINs and acetylcholine release in the dorsomedial striatum. Despite the presence of prominent projections from the primary visual cortex and other sensory cortices to the dorsal striatum, these cortical areas do not connect strongly to striatal CINs and are unable to drive dopamine release. In contrast, frontal cortical areas such as the prelimbic and anterior cingulate cortices robustly recruit striatal CINs and acetylcholine to drive dopamine release *ex vivo* and *in vivo*. Thus, this study identifies a key distinction between primary sensory and frontal cortical inputs to the striatum, with important implications for how the brain processes behaviorally relevant stimuli that support associative and reinforcement learning.

Previous studies showed that certain cortical and thalamic inputs can evoke striatal dopamine *ex vivo*, but two key questions remained: Which cortical areas selectively recruit CINs to regulate dopamine? Does this mechanism operate *in vivo* under behaviorally relevant conditions? Initial slice studies demonstrated that inputs from the motor cortex and parafascicular thalamus strongly engaged CINs in the dorsal striatum and triggered dopamine-dependent plasticity^19^. The retrograde monosynaptic rabies tracing from our study supports these findings and identifies the dorsolateral and dorsocentral striatum as the target of the motor cortex and parafascicular thalamus inputs to CINs. Subsequent studies extended the findings to prelimbic cortex and intralaminar thalamus inputs^22^; our anatomical and functional experiments confirm those findings and further identify the dorsomedial subregion as the target from these PL/ACA inputs to striatum. *In vivo* stimulation of the prelimbic cortex was shown to increase striatal glutamate, acetylcholine, and dopamine^22^, though with insufficient temporal precision to link dopamine release to behavioral events. Our results address this gap by providing *in vivo* evidence that frontal cortical areas (PL/ACA) exert temporally precise control over striatal dopamine. Crucially, this ability to drive dopamine via CINs is restricted to specific frontal cortical and thalamic inputs and we show that it is absent in primary sensory cortices.

The most parsimonious explanation for why frontal, but not posterior sensory, cortices can regulate striatal dopamine release, is the differential connectivity of cortical areas to striatal CINs. However, a caveat of these *in vivo* experiments is that optogenetic stimulation of cortical axons in the striatum might also activate axon collaterals that synapse onto midbrain dopamine neurons. PL/ACA projections to the dorsal striatum also send collaterals to dopaminergic midbrain regions, which could contribute to the striatal dopamine signals we measured *in vivo*^44^. While it is difficult to rule out this possibility, our *ex vivo* experiments confirm that PL/ACA projections form strong synapses onto CINs and robustly recruit them to evoke local dopamine release, consistent with a direct cortical effect on striatal dopamine transmission. Because all axons from neurons projecting to the striatum are severed in *ex vivo* brain slice preparations, only the axon segments within the slice are activated during the experiments. Any direct projections from cortex to the midbrain dopamine neurons are excluded and cannot be responsible for the dopamine signals measured *ex vivo*. Thus, although collateral activation of cortical-midbrain projection may contribute *in vivo*, especially during longer stimulations that might recruit larger circuits, direct recruitment of CINs also contributed to local regulation of dopamine release, as seen with both single pulses of stimulation and short train stimulation of the frontal cortex. We conclude that the unique ability of PL/ACA to evoke dopamine likely arises from the strength of its connections with CINs, a conclusion supported by both anatomical and functional evidence.

In considering our anatomical evidence, it is important to note that rabies tracing results vary with the striatal location of the starter cells. Here, we targeted the anterior dorsal striatum and observed a medial-to-lateral segregation of inputs to CINs. Different patterns would be expected for starter cells in the striatal tail or nucleus accumbens. The anterior dorsal striatum integrates frontal, motor, and sensory signals and is a key hub for goal-directed and habitual behavior. Future studies should assess whether similar connectivity rules apply in the nucleus accumbens or posterior striatum, and how these differences might influence local microcircuits and behavior. While rabies tracing identifies the anatomical distribution and laminar origin of inputs to CINs, it cannot quantify the functional efficacy of the connections. For this, we use electrophysiology, which provides functional measures of synaptic strength. Whole-cell recordings showed that although CINs receive inputs from barrel and visual cortices, these synapses were too weak to drive them independently. It remains possible, however, that coincident activation from multiple sensory areas could summate to evoke dopamine release. Broadly, the functional results aligned with the anatomical data: rabies tracing revealed that CINs in the dorsomedial striatum (DMS) receive dense input from frontal regions, particularly ACA, whereas CINs in the dorsolateral striatum (DLS) are more strongly innervated by motor and somatosensory cortices. This organization parallels the connectivity of cortical inputs to striatal MSNs^45–47^, reinforcing the view that the DMS is engaged in cognition and goal-directed behavior, while the DLS supports action selection and habits^3,48–50^. Our tracing data refine previous work by detailing the cortical and thalamic sources of input to CINs within specific striatal subregions using an improved ChAT-IRES-Cre mouse line without common events of spontaneous recombination^51^.

Our results provide direct support for the idea that cortical inputs to the striatum can evoke dopamine release through a local circuit involving CINs, but the relationship between dopamine and acetylcholine is complex and remains controversial. Two studies showed that striatal acetylcholine and dopamine signals fluctuate in an anti-correlated manner *in vivo*^35,36^. These observations were further supported by recent findings from *ex vivo* slice experiments showing acetylcholine may, in some contexts, inhibit midbrain-evoked dopamine^23^, which could explain the anticorrelation observed in the *in vivo* signals. One of these studies reported that intracranial infusions of DHβE, a nicotinic receptor blocker, did not affect spontaneous dopamine signals, nor reward-driven signals^35^, which midbrain neurons would be expected to drive.

Meanwhile, the other study saw that inhibiting acetylcholine release from CINs, striatum-wide, had significant effects on behavior and behaviorally evoked dopamine signals^36^. However, more local inhibition of acetylcholine release in the ventrolateral striatum had no effect in the same contexts^36^. These studies did not examine sensory cue-evoked responses nor the role of acetylcholine-mediated control over dopamine throughout learning. Given the historic role of CIN firing pattern in cue responses and learning, these are important gaps to fill.

More recent work has shown compelling evidence that the local microcircuitry regulating striatal dopamine signals comes into play during both effortful tasks (dorsal striatum)^32^ and active avoidance behavior (nucleus accumbens)^52^. These findings are in agreement with our identification of the proprietary role of frontal cortical areas in driving the acetylcholine-dependent dopamine signals. This conclusion has further behavioral implications that need to be pursued. For example, future studies can identify the temporal dynamics of this mechanism and how it integrates with midbrain-evoked dopamine. A deeper understanding is needed to sort out the contribution of CIN-dependent dopamine release to the dopamine tone *in vivo*.

In summary, our study reveals that sensory stimuli and frontal cortical regions, including the prelimbic and anterior cingulate cortices, robustly engage CINs and acetylcholine to trigger striatal dopamine signals in the anterior striatum. Primary sensory cortices, while projecting to the same striatal subregion, don’t recruit CINs nor share the ability to drive striatal dopamine. Thus, we reveal a key distinction between sensory and frontal cortical inputs, identifying the latter as privileged drivers of striatal dopamine that may support associative and reinforcement learning. Disentangling the mechanisms behind cholinergic control over striatal dopamine and associated learning could prove critical to understanding the root cause of disorders involving dysregulation of striatal dopamine.

## Materials and Methods

### Animals

All experimental procedures were approved by the NIH Institutional Animal Care and Use Committee (IACUC) and complied with Public Health Service policy on the humane care and use of laboratory animals. Both female and male mice were used for all experiments. Electrophysiology experiments used either a ChAT-IRES-Cre (Δneo) line^53^ (for CIN or MSN recordings, B6.129S-*Chat*^*tm1(cre)Lowl*^/MwarJ; RRID: IMSR_JAX:031661), a Pvalb-IRES-Cre line (for MSN recordings, B6.129P2-Pvalbtm1(cre)Arbr/J; RRID: IMSR_JAX:017320), or a SST-IRES-CRE line^54^ (for MSN recordings, B6J.Cg-Ssttm2.1(cre)Zjh/MwarJ; RRID: IMSR_JAX:028864) crossed with an Ai14 Cre-dependent tdTomato line^55^ (B6.Cg-*Gt(ROSA)26Sor*^*tm14(CAG-tdTomato)Hze*^/J; RRID: IMSR_JAX:007914). Fast-scanning cyclic voltammetry experiments used wild-type C57B6/J mice or the ChAT-IRES-Cre (Δneo) x Ai14 cross. Rabies experiments were done in the ChAT-IRES-Cre (Δneo) line. ChAT-IRES-Cre (Δneo), C57BL6/J, and Drd1a-Cre (B6.FVB(Cg)-Tg(Drd1-cre)EY262Gsat/Mmucd, RRID: MMRRC_030989-UCD) mice were used for *in vivo* fiber photometry experiments.

### Fiber photometry

#### Surgeries

Animals (N = 27, 16 males, 11 females; age: 3 – 9 months, average 5 months, Table S1) were injected with AAV9-hSyn-dLight1.3b^56^ (titer = 2.5E+13; Addgene #135762-AAV9; RRID:Addgene_135762), AAV9-Syn-FLEX-GCaMP8s-WPRE^57^ (titer =2.7E+13, Addgene #162377-AAV9; RRID: Addgene_162377 ), AAV9-Syn-FLEX-GCaMP8f-WPRE^57^ (titer = 2.0E+13, Addgene #162379-AAV9, RRID: Addgene_162379) and/or AAV9-Syn-GRAB-ACh3.0(4.3)^58^ (titer =6.7E+12, WZ Bioscience #YL001003-AV9-PUB) in DMS (400–500 nL at 180 nL / min; +0.9 mm AP, +/− 1.3 mm ML, −2.8 mm DV from Bregma). A subset of these mice were additionally injected with AAV5-hSyn-ChrimsonR-tdTomato^59^ (titer = 1.20E+13; Addgene #59171-AAV5; RRID:Addgene_59171) in prelimbic cortex (120 nL at 120 nL / min; +2.1 mm AP, +/− 0.4 mm ML, −2.3 mm DV from Bregma) or anterior cingulate cortex (120 nL at 120 nL / min; +1.0 AP, +/− 0.3 mm ML, −1.5 mm DV from Bregma). A custom headpost and optic fiber cannulae (400um Ø, 0.37 NA, 2.5mm long; Doric) were implanted above the injection sites using Metabond (C&B Parkell).

#### Photometry system

Thorlabs LED drivers (LEDD1B) were used to drive Thorlabs 405 nm (M405F1), 470 nm (M470F4), and 625 nm (M625F2) fiber-coupled LEDs connected to a six-channel Doric filter cube (FMC6_IE(400–410)_E1(460–490)_F1(500–540)_E2(555–570)_F2(580–680)_S) by 0.5 NA / 400 μm Ø patch cords (Thorlabs, M301L01). Subject cords were custom-ordered from Doric (MFP_400/440/1100–0.37_1m_FCM-ZF1.25(FP)_LAF). Newport Femtowatt Receivers (Doric, NPM_2151_FOA_FC) were connected to the filter cubes using custom 0.5 NA / 600 μm Ø patch cords (Thorlabs). Femtowatt receivers fed into a Tucker Davis Technologies RZ5P processor. All fiber optic cords were photobleached for 24 hours before the start of the experiment. 405 nm and 470 nm LEDs were driven at 215 and 326 Hz. Data were collected at 1017 Hz and demodulated using Tucker Davis Technologies Synapse software. An Arduino interfaced with the 625 nm LEDs and the RZ5P processor controlled and timestamped optical stimulation.

#### Data collection

405 nm and 470 nm LEDs were calibrated daily to 20 μW and 30 μW, respectively, as measured at the fiber tip. Mice were head-fixed in an acrylic tube for the duration of recordings. Mice were headfixed into a custom head fixation setup with their body resting within an acrylic tube. All behavioral tasks were performed in a dark sound-attenuated behavior chamber (Med Associates) fitted with 20mm black sound-insulating foam. All behavioral tasks and external stimuli were controlled using pyControl^60^ (Open Ephys Production Site).

Auditory cues were delivered at 7.5 KHz at 70 dB for 500 msec. Tones were delivered via an 8 Ohm speaker (DigiKey; part number GF0401M-ND) placed 6 inches directly above the mouse’s head. The speaker was connected to a microcontroller (ATmega328P) where tones were generated using Arduino’s tone function. Visual cues were delivered via a pyControl houselight LED that uses three 16 lux LEDs on a plastic strip, placed in the behavior chamber above / slightly in front of the mouse. The luminance of the LED was measured to be 5.9 cd/m^2^ on the floor of the box, and 0.6 cd/m^2^ on the black foam sidewalls.

625 nm LEDs used for optogenetic stimulation were similarly calibrated to 5.5 mW. Optogenetic stimulation was delivered every 30 seconds in either single 10 ms pulses, 10 ms three-pulse trains at 20 Hz, or 10 ms ten-pulse trains at 20 Hz.

#### Pharmacology

Mecamylamine (2843/10, Tocris) was prepared fresh in 0.9% sterile saline for a final concentration of 10 mg / 10 mL, allowing mice to be injected with a 10 mL / kg dosage. During pharmacology sessions, 15 minutes of optogenetic data were collected followed by 5 repetitions of each cue. Mice were then injected intraperitoneally with mecamylamine or 10 mL / kg saline and returned to their home cage for 30 minutes before repeating the optogentic stimulation and sensory cue protocols.

#### Analysis

Data were converted to ΔF/F signals using GuPPy, an open-source Python package^61^ (RRID: SCR_02235345). The same Python package was used to baseline-correct the traces and create PSTHs. The preprocessed data were then further analyzed and plotted in Python. Data were z-scored either over a single file (baseline non–pharmacological sessions) or over the “pre” and “post” files together (pharmacological sessions). Baseline data were averaged across sessions, within each hemisphere. Pharmacological data were always collected and reported from a single session.

#### Histology

Mice were deeply anesthetized with isoflurane and transcardially perfused with phosphate-buffered saline, followed by 4% paraformaldehyde (PFA). Brains were extracted, post-fixed in 4% PFA at 4 °C overnight, and transferred to 30% sucrose in PBS for >48 h at 4 °C. Brains were flash frozen in isopentane and stored at −80 °C. 35-μm coronal cryo-sections were cut using a cryostat and collected in PBS containing 0.01% sodium azide. To verify viral expression and fiber placement, brain sections were gently rocked for 3 × 10 min in PBS, blocked with 10% normal goat serum (NGS; Jackson Immuno, 005-000-121; RRID: AB_2336990) in PBS containing 0.1% Triton X-100 (PBS-Tx) for 1 h at room temperature, and then incubated in rabbit anti-GFP, 1:500 (Invitrogen, G10362; RRID: AB_2536526) primary antibody in PBS-Tx at 4 °C for 24 h. Sections were rinsed 3 × 10 min with PBS and incubated in 488 anti-rabbit,1:800 (Jackson Immuno, 111-545-003; RRID: AB_2338046) secondary antibody in PBS-Tx for 2 h at room temperature. Sections were washed 3 × 10 min with PBS, mounted on slides, and coverslipped with ProLong^™^ Gold antifade reagent with DAPI (Invitrogen, P36935). All slides were imaged using consistent exposure settings on a ZEISS Axioscan 7 slide scanner or a Keyence BZ-X810 microscope.

### Slice physiology

#### Surgeries

Animals (n = 47; 21 males, 26 females; age: 2–13 months, median 3 months) were injected with AAV5-hSyn-ChR2(H134R)-eYFP (titer = 1.80E+13; Addgene, #26973; RRID:Addgene_26973) and/or AAV5-hSyn-ChrimsonR-tdTomato (as above). PL injections (100–120 nL at 120nL/min) were targeted to +2.1 mm AP, +/− 0.4 mm ML, −2.3mm DV from Bregma. ACA injections (100–120 nL at 120nL/min) were targeted to +1.0 mm AP, +/− 0.3 mm ML, −1.5mm DV from Bregma. Somatosensory cortex injections (120 nL at 120nL/min) were targeted to −1.25 mm AP, +/− 3.0 mm ML from Bregma, and −0.5 mm DV from Pia. Visual cortex injections (120 nL at 120nL/min) were targeted to −3.5 mm AP, +/− 2.3 ML from Bregma, and −0.5 mm from Pia. Auditory cortex injections (120 nL at 120nL/min) were targeted to −2.7 mm AP, +/− 4.3 mm from Bregma, and −0.7 mm from Pia.

#### Slice preparation

Mice were anesthetized and decapitated at least four weeks post-surgery. Brains were extracted, mounted on a vibratome (VT-1200S, Leica Microsystems), and sliced coronally (240 μm) in oxygenated cutting solution heated to 32°C, containing the following (in mM): 90 sucrose, 80 NaCl, 24 NaHCO3, 1.25 NaH2PO4, 10 glucose, 3.5 KCl, 0.5 CaCl2, 4.5 MgCl2, and 3 kynurenic acid. Slices were incubated for 20 min at 32°C in ACSF containing the following (in mM): 124 NaCl, 1 NaH2PO4, 2.5 KCl, 1.3 MgCl2, 2.5 CaCl2, 20 glucose, 26.2 NaHCO3, and 0.4 ascorbic acid, and kept at room temperature after that until use. The recording chamber was perfused at 2 ml/min with ACSF heated to 32°C using an inline heater (Harvard Apparatus).

#### Fast scanning-cyclic voltammetry

FSCV was performed in the dorsal striatum. Carbon-fiber electrodes (CFEs) were prepared with a cylindrical carbon fiber (7 μm diameter, ~150 μm of exposed fiber) inserted into a glass pipette. Before use, the CFEs were conditioned with an 8-ms-long triangular voltage ramp (−0.4 to 1.2 and back to −0.4 V vs Ag/AgCl reference at 400 V/s) delivered every 15 ms. CFEs showing current >1.8 μA or <1.0 μA in response to the voltage ramp at ~0.6 V were discarded. CFEs were held at −0.4 V versus Ag/AgCl, and the same triangular voltage ramp was delivered every 100 ms. Using the same CFE and location, dopamine signals were evoked by alternating electrical and optical stimulation, delivered every 2 mins. For electrical stimulation, a glass pipette filled with ACSF was placed near the tip of the carbon fiber and a rectangular pulse (0.2 ms, 400 μA). For optogenetic stimulation, 470 nm blue light (1.3 mW) was delivered through either a 40x objective via a CoolLED (pE-800) or a fiber-optic patch cord (200 μm diameter, 0.22 NA, ThorLabs) attached to a ThorLabs fiber-coupled LED (M470F4, driven by a LEDD1B driver). The light source was placed over the CFE to deliver square light pulses (5–10ms). Data were collected with a retrofit headstage (CB-7B/EC with 5 mΩ resistor) using a Multiclamp 700B amplifier (Molecular Devices) after a low-pass filter at 3 kHz and digitized at 100 kHz using a data-acquisition board (NI USB-6229 BNC, National Instruments). Data acquisition and pre-processing were performed using custom-written software, VIGOR, in Igor Pro (Wavemetrics) using mafPC (courtesy of MA Xu-Friedman). Further analysis was conducted using Python. The current peak amplitudes of the evoked dopamine transients were converted to dopamine concentration according to a post-experimental calibration using 1–3 μm DA.

#### Electrophysiology

Striatal CINs were identified by fluorescence and confirmed by their characteristic spontaneous firing pattern. Whole-cell recordings were performed from CINs in the striatum using glass pipette electrodes with a resistance of ~3–4 MΩ, filled with an internal solution (pH 7.25, 290–310 mOsm) containing the following (in mM): 120 CsMS, 10 CsCl, 10 HEPES, 0.2 EGTA, 10 sodium phosphocreatine, 4 Na2-ATP, and 0.4 Na-GTP. For input-output curves, internal solution also contained 4.4 mM lidocaine to block sodium channels and prevent firing. Cholinergic interneurons were held at a holding potential of −70 mV to keep these spontaneously firing neurons far away from firing threshold. Medium spiny neurons were held at a holding potential of −70 mV or −55 mV, where the cell input resistance is higher. All recordings were done in the presence of 5 μM CPP (Tocris, #0247) to block NMDA currents. Excitatory postsynaptic currents were recorded in response to 1 or 10 ms duration square light pulses (titrated based on synapse strength to avoid inducing action potentials). A dual-color LED (Doric LEDC2_405/595) was used to alternate between pulses of violet-shifted (405 nm, 1.6 mW) or orange light (595 nm, 1.0 mW). Data were collected using a Multiclamp 700B amplifier after a low-pass filter at 1 kHz and digitized at 5 kHz using pClamp10 software (Molecular Devices).

#### Analysis

Data were analyzed in Python using pyABF (https://github.com/swharden/pyABF). Sweeps were filtered with a 100Hz 4th-order lowpass Buttworth filter to remove high-frequency noise. Data were baseline corrected to a standard window prior to stimulation, and noise was subtracted to the height of the average peak within that window. Peaks were detected as the minimum (EPSCs) within a 300 ms window from stimulation. For visualization, the absolute values of the EPSCs were plotted as positive numbers. All data are presented as the mean and SEM.

#### Anatomical tracing

ChAT-IRES-Cre (Δneo) (n = 12, 7 males, 5 females; age: 2–21 months, median 2 months) were injected unilaterally in DMS or DLS, as described above, with helper viral vector AAV8-DIO-Ef1a-TVA-Flag-2A-N2cG (Addgene #172360-AAV8; RRID:Addgene_172360), and rabies retrograde vector EnVA-CVS-N2c-tdTomato-FlpO one week apart^62^. Six days after the second injection, mice were deeply anesthetized with isoflurane and transcardially perfused with phosphate-buffered saline, followed by 4% paraformaldehyde (PFA). Brains were removed, fixed overnight in 4% PFA and transferred to 20% sucrose in PBS. After the brains had equilibrated in 20% sucrose/PBS coronal sections were cut at 50 μm on a freezing microtome and collected in PBS. TdTomato labeling in trans-synaptically rabies labeled neurons was amplified immunohistochemically using sequential incubation in primary antibodies, rabbit anti-RFP (Rockland, 600-401-379) followed by secondary antibodies goat anti-rabbit Alexa Fluor 555 (ThermoFisher, A32732). Sections were mounted onto slides and labeled with the fluorescent blue Nissl stain Neurotrace 435 (ThermoFisher, N21479). All sections through the brain from the frontal pole to the brainstem were mounted sequentially onto slides (96 sections/mouse) and imaged at 10x (Zeiss PlanNeoFluor objective, 10x NA0.3) using a Zeiss AxioImagerM2 fluorescence microscope with an OrcaFlash4.0 camera and Ludl motorized 2axis stage controlled by Neurolucida software (MBF Bioscience, Williston, VT). Each 50 μm sections was imaged in 7 μm steps, and the tiled images were then compiled into a single image and collapsed into a single plan using the DeepFocus function in Neurolucida. Analysis of labeled neurons by reconstructing the series of serial sections into a whole brain volume, detecting and marking labeled neurons and registering the location of neurons labeled with tdTomato into the Allen Brain Atlas using NeuroInfo Software^63^. To display the brain wide distribution of cortical neurons providing synaptic inputs to striatal ChAT neurons coordinates of presynaptic rabies labeled cortical neurons registered to the Allen CCF are projected along curved streamlines of the cortex to a flattened mapping of the cerebral cortex^64^. Data were further analyzed in Python and are presented as a percentage of the total labeled cells in each overall region (cortex, thalamus, midbrain, and basal ganglia).

## Supplementary Material

Supplement 1
