# Supplementary material for: Cholinergic-dependent dopamine signals in mouse dorsal striatum are regulated by frontal but not sensory cortices
Source: bioRxiv. 2025 Sep 30:2025.09.30.679538. Preprint. [Version 1] doi: 10.1101/2025.09.30.679538 (PMC12622003; doi:10.1101/2025.09.30.679538)
Supplement: Supplement 1 [file media-1.pdf]

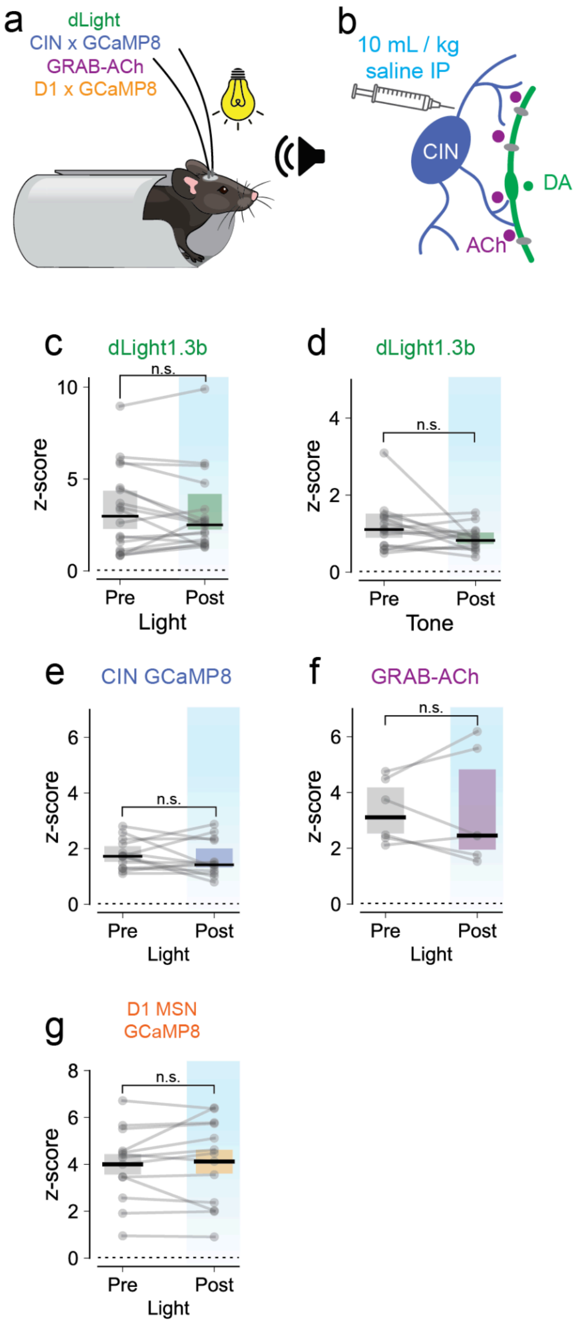
a
dLight
CIN x GCaMP8
GRAB-ACh
D1 x GCaMP8
b
10 mL / kg
saline IP
CIN
DA
ACh
c
dLight1.3b
n.s.
z-score
Pre
Post
Light
d
dLight1.3b
n.s.
z-score
Pre
Post
Tone
e
CIN GCaMP8
n.s.
z-score
Pre
Post
Light
f
GRAB-ACh
n.s.
z-score
Pre
Post
Light
g
D1 MSN
GCaMP8
n.s.
z-score
Pre
Post
Light

Supplemental Figure 1: Saline injection does not affect sensory cue-evoked striatal signals

a-b. Implanted mice expressing dLight1.3b, GCaMP8 in CINs or D1-MSNs, or GRAB-ACh were headfixed and presented with 500 ms lights or tones. To control for any effects of receiving an injection, mice were presented with these stimuli before and after receiving a 10 mL/kg injection of 0.9% sterile saline.

c-d. Saline effects on light-evoked and tone-evoked dopamine responses (n = 18 hemispheres from 14 mice, p = 0.67 and n = 15 hemispheres from 11 mice, p = 0.09, respectively, Wilcoxon Signed Rank test).

e-g. Saline effects on light-evoked CIN GCaMP8 responses (n = 13 hemispheres from 11 mice, p = 0.50, Wilcoxon Signed Rank test), on light-evoked GRAB-ACh responses (n = 6 hemispheres from 6 mice, p = 1.0, Wilcoxon Signed Rank test), and D1 MSN GCaMP8 responses (n = 13 hemispheres from 8 mice, p = 0.45, Wilcoxon Signed Rank test). For all panels, “n.s.” denotes no significant difference.

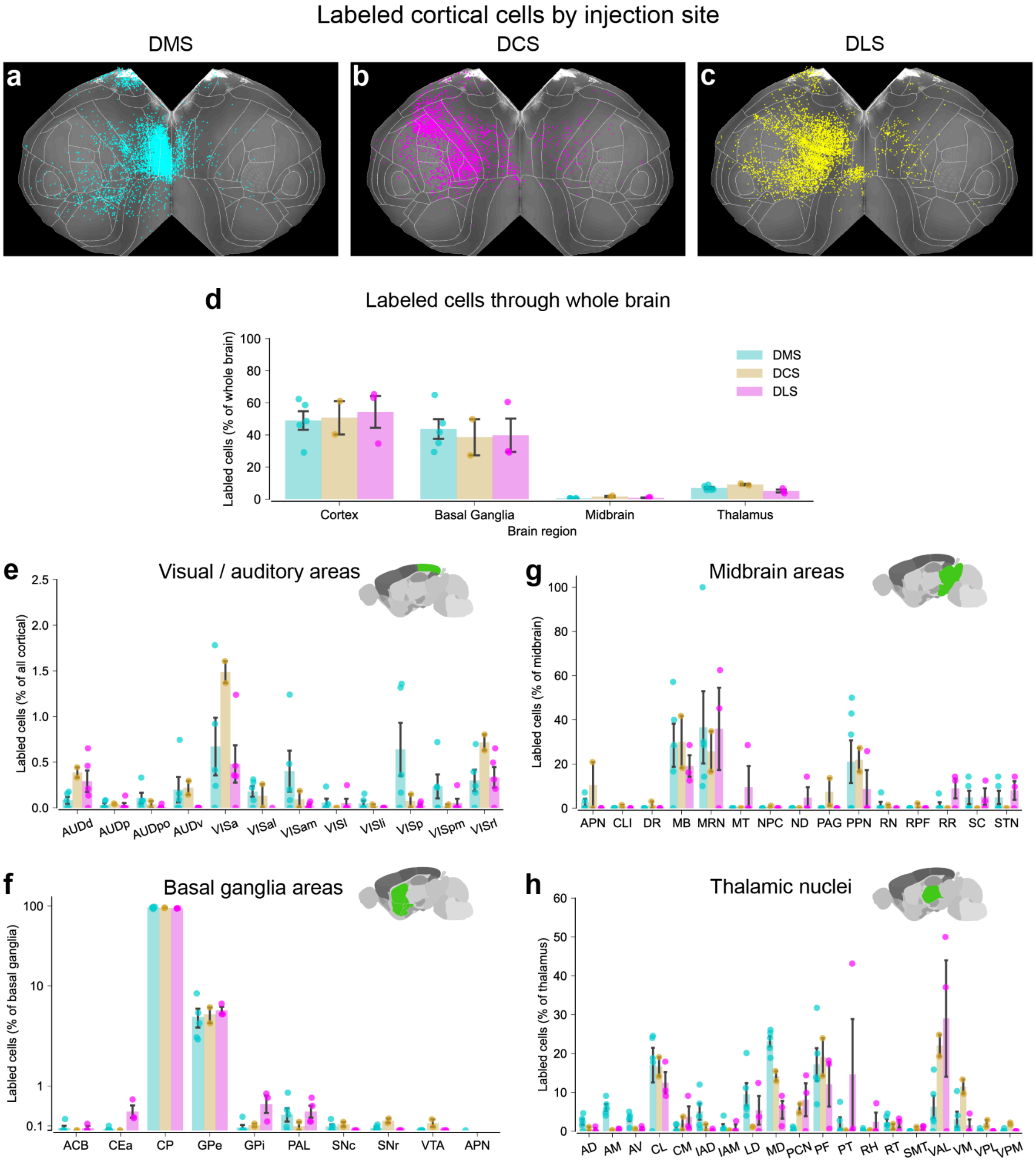

Labeled cortical cells by injection site
DMS
DCS
DLS
a
b
c
d
Labeled cells through whole brain
Labled cells (% of whole brain)
DMS
DCS
DLS
Cortex
Basal Ganglia
Midbrain
Thalamus
Brain region
e
Visual / auditory areas
Labled cells (% of all cortical)
AUDd
AUDp
AUDpo
AUDv
VISa
VISal
VISam
VISl
VISli
VISp
VISpm
VISrl
g
Midbrain areas
Labled cells (% of midbrain)
APN
CLI
DR
MB
MRN
MT
NPC
ND
PAG
PPN
RN
RPF
RR
SC
STN
f
Basal ganglia areas
Labled cells (% of basal ganglia)
ACB
CEa
CP
GPe
GPi
PAL
SNc
SNr
VTA
APN
h
Thalamic nuclei
Labled cells (% of thalamus)
AD
AM
AV
CL
CM
IAD
IAM
LD
MD
PCN
PF
PT
RH
RT
SMT
VAL
VM
VPL
VPM

Supplemental Figure 2: Retrograde rabies tracing of monosynaptic projections to CINs - regional labeling

a-c. Top-down whole brain views of presynaptic rabies labeled cortical neurons providing inputs to CINs in DMS (cyan, left), DCS (yellow, center), and DLS (magenta, right).

d. Labeled cells across the cortex, basal ganglia, thalamus, and midbrain as a percentage of all cells labeled throughout the brain. Regardless of injection site, expression was more widespread across the cortex and basal ganglia than in the midbrain or thalamus.

e. Cells labeled in primary and secondary visual and auditory cortices, as a percentage of all labeled cortical cells. Only a very small percentage of labeled cells (0.5-1.5%) were found in these regions. However, it should be noted that more labeling was found in some secondary visual areas (VISa, VISrl) than in the primary visual cortex.

f. Percent of labeled cells within basal ganglia subregions, as a percent of all labeled cells in basal ganglia. Note that the y-axis is log-scaled. Most labeled cells were located locally within the striatum (CP, caudate-putamen), with additional labeling of the globus pallidus (GPe, GPi). Lesser expression was found throughout the rest of the basal ganglia (ACB, PAL, SNc, SNr, ACB, VTA, CEa).

g. Labeled cells within the midbrain, by subregion, as a percentage of all labeled cells in the midbrain. Labeling in the midbrain was sparse; however, most labeling was seen in MRN, PPN, and other unspecified midbrain areas (MB).

h. Percent of labeled cells within the thalamus by subregion, as a percentage of all labeled cells in the thalamus. Injections across the dorsal striatum produced labeling in CL and PF, while medial injections produced more labeling in MD, and lateral injections produced more labeling in PCN and VAL. For all panels, the area name and abbreviation follow Allen Institute naming conventions (**Table S2**).

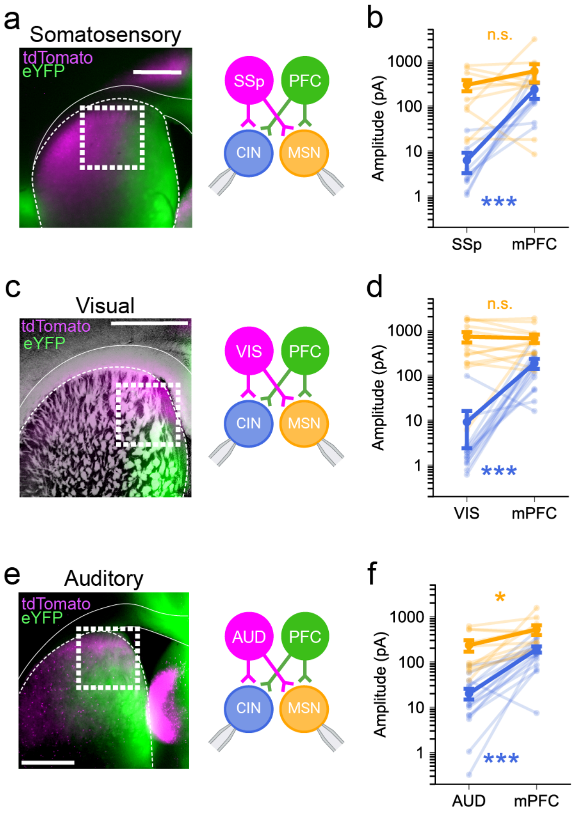

Supplemental Figure 3: Paired whole-cell voltage clamp data

To directly compare how single cells respond to input from sensory areas and frontal areas, we expressed in the same animal ChrimsonR-tdT in a sensory area and ChR2-eYFP in the prelimbic cortex. This allowed us to record from striatal cells in areas of overlapping expression and measure synaptic responses evoked by 405 nm violet light to drive ChR2-expressing terminals and 590 nm light to drive Chrimson-expressing terminals. The opsin's selection was made with the intention of minimizing false positive responses when stimulating sensory cortex axons. ChR2 is activated by blue light, which can also spuriously activate ChrimsonR. Red light, however, provides cleaner activation of only ChrimsonR. Therefore, we expressed ChR2 in prelimbic terminals and ChrimsonR in sensory terminals. This way, red light activation of ChrimsonR would exclusively activate sensory terminals. If the expression were swapped, blue light stimulation of sensory terminals would also stimulate the stronger projections from the prelimbic cortex, resulting in false positives.

a, c, e. Coronal brain sections from ChAT-tdTomato reporter mice expressing ChR2-eYFP in PL (green) and ChrimsonR-tdTomato in either SSp (a), VISp (c), or AUDp (e, magenta). Dual opsin optogenetic stimulation was used to probe for evoked synaptic responses while recording from striatal CINs (blue) and MSNs (orange) in areas of axonal overlap (white squares). Excitatory synaptic responses were measured using whole-cell voltage-clamp electrophysiology. All white scale bars are 1 mm.

b, d, f. Mean amplitude of EPSCs recorded from CINs (blue) and MSNs (orange) following optogenetic stimulation of PL and sensory corticostriatal projections. Dark dots represent population mean ± SEM, and light dots represent data from each cell recorded.

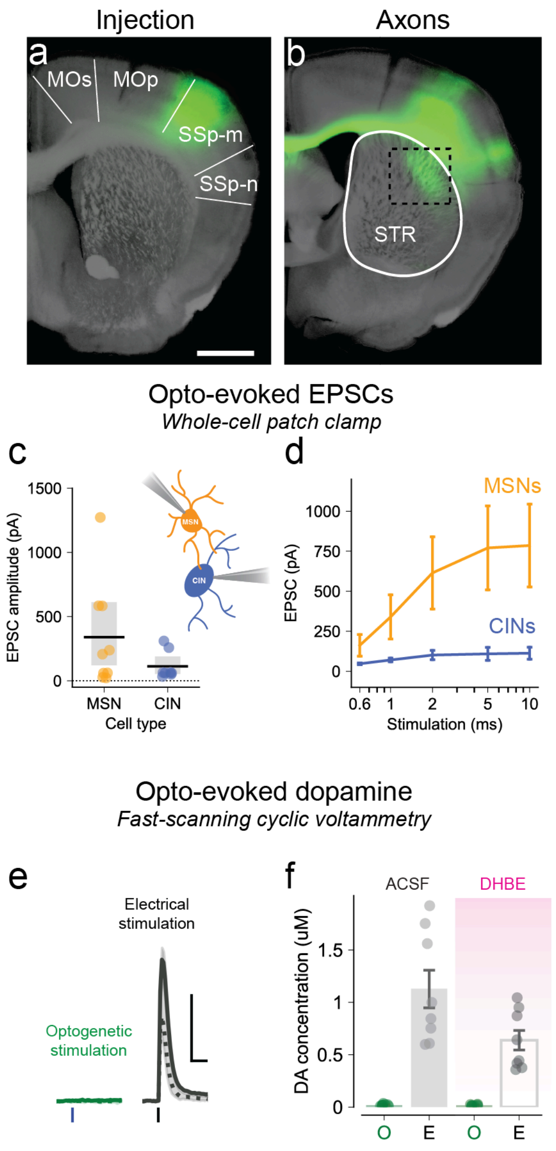

Supplemental Figure 4: SSp-m terminals do not drive striatal CINs strongly enough to evoke dopamine

Retrograde rabies tracing (Figure 3) from CINs in the DLS produced a reasonable percentage of labeled cells in the mouth area of somatosensory cortex (SSp-m). We therefore sought to test whether these synapses are strong enough to drive striatal CINs and striatal dopamine release.

a. ChR2-eYFP was injected into SSp-m bilaterally in three ChAT-IRES-cre x tdTomato mice. Image shows the injection site (SSp-m, a). Scale bar is 1 mm.

b. Whole-cell voltage clamp electrophysiology and fast-scan cyclic voltammetry were carried out in DLS, with optogenetic stimulation of SSp-m terminal (black box).

c-d. Optogenetic stimulation of SSp-m terminals produced measurable EPSCs from MSNs (orange) and rare measurable responses from CINs (blue, 2 out of 9 cells). Increasing the stimulation duration dramatically increased the responses measured from MSNs, but only slightly increased the responses measured from CINs (**d**).

e. Optogenetic stimulation of SSp-m terminals failed to produce measurable dopamine transients, despite large electrically-evoked transients recorded from the same sites.

f. Average dopamine transient amplitudes elicited by electrical stimulation (gray bars) and optogenetic stimulation. Each dot represents data from one slice. Pink-shaded data were collected after DHβE bath application.

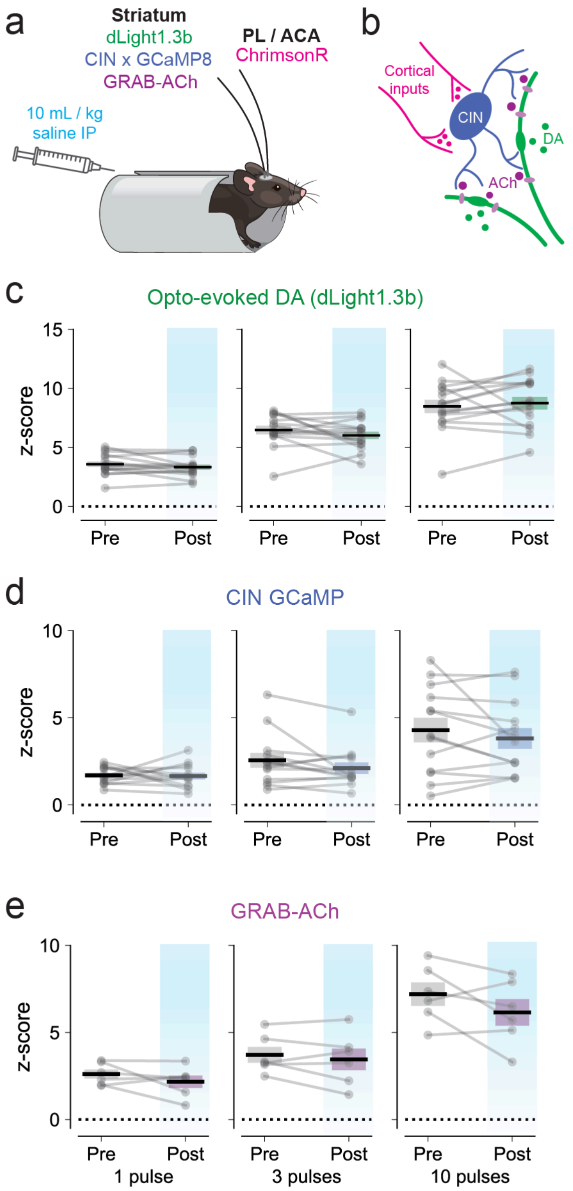

Supplemental Figure 5: Saline injection does not affect optogenetically evoked striatal responses *in vivo*

a-b. Implanted mice expressing dLight1.3b, GCaMP8 in CINs, or GRAB-ACh in DMS were headfixed and received optogenetic stimulation of PL / ACA striatal terminals (see Figure 6, Methods). To control for any effects of receiving an injection, mice received this stimulation before and after receiving a 10 mL/kg injection of 0.9% sterile saline.

c-e. Saline effects on optogenetically-evoked dopamine responses (e), CIN GCaMP8 responses (d), and GRAB-ACh responses (e). Comparisons of pre- and post-injection were not significant (dLight1.3b, left to right: $p = 0.56$, $p = 0.45$, $p = 0.19$; GCaMP8s, left to right: $p = 0.84$, $p = 0.15$, $p = 0.50$; GRAB-ACh, left to right: $p = 0.44$, $p = 0.56$, $p = 0.31$; Wilcoxon Signed Rank test).

Supplemental Table 1: Fiber photometry animals & viral expression

Mouse	Sex	Age (m)	Genotype	Left Hem.	Right Hem.	Chrimson
**HG-04**	M	4	ChAT-Cre		GCaMP8s	PL
**HG-05**	M	6	ChAT-Cre		GCaMP8s	PL
**HG-06**	M	7	ChAT-Cre	ACh3.0	GCaMP8s	PL
**HG-08**	**F**	3	ChAT-Cre	ACh3.0	GCaMP8s	PL
**HG-09**	**F**	3	ChAT-Cre	ACh3.0	GCaMP8s	PL
**HG-10**	**F**	3	ChAT-Cre	ACh3.0	GCaMP8s	PL
**HG-11**	**F**	6	ChAT-Cre	GCaMP8f	dLight1.3b	PL
**HG-12**	**F**	6	ChAT-Cre	GCaMP8f	dLight1.3b	PL
**HG-14**	M	6	ChAT-Cre	GCaMP8f	dLight1.3b	PL
**HG-15**	**F**	3	ChAT-Cre	GCaMP8f	GCaMP8f	ACA
**HG-16**	**F**	3	ChAT-Cre	GCaMP8f	GCaMP8f	ACA
**HG-17**	M	3	ChAT-Cre	GCaMP8f	dLight1.3b	ACA
**HG-18**	M	5	WT	ACh3.0	dLight1.3b	ACA
**HG-19**	**F**	5	WT	ACh3.0	dLight1.3b	ACA
**HG-20**	M	4	WT	ACh3.0	dLight1.3b	ACA
**RR-03**	M	4	WT	dLight1.3b	dLight1.3b	PL
**RR-05**	M	4	WT	dLight1.3b	dLight1.3b	PL
**RR-07**	M	4	WT	dLight1.3b	dLight1.3b	PL
**RR-09**	M	4	WT	dLight1.3b	dLight1.3b	PL
**LA-11**	M	7	D1-Cre	GCaMP8s	GCaMP8s	
**LA-12**	M	7	D1-Cre	GCaMP8s	GCaMP8s	
**LA-13**	M	7	D1-Cre	GCaMP8s	GCaMP8s	
**LA-15**	M	9	D1-Cre	GCaMP8s	GCaMP8s	
**LA-16**	M	9	D1-Cre	GCaMP8s	GCaMP8s	
**LA-18**	**F**	8	D1-Cre	dLight1.3b	GCaMP8s	
**LA-19**	**F**	8	D1-Cre	dLight1.3b	GCaMP8s	
**LA-20**	**F**	8	D1-Cre	GCaMP8s	dLight1.3b	

Supplemental Table 2: Allen Institute Brain Region Nomenclature

**Abbreviation**	
**ACA**	Anterior Cingulate Area
**ACB**	Accumbens
**AD**	Anterodorsal Nucleus
**AI**	Agranular Insular Area
**AM**	Anteromedial Nucleus
**APN**	Anterior Pretectal Nucleus
**AUDp**	Primary Auditory Area
AUDd	*Dorsal Auditory Area*
AUDpo	*Posterior Auditory Area*
AUDv	*Ventral Auditory Area*
**AV**	Anteroventral Nucleus
**BG**	Basal Ganglia
**CEa**	Central Amygdalar Nucleus
**CL**	Claustrum
**CLI**	Central Linear Nucleus Raphe
**CM**	Central Medial Nucleus
**CP**	Caudoputamen
**CTX**	Cortex
**DR**	Dorsal Nucleus Raphe
**ECT**	Ectorhinal Area
**GPe**	Globus Pallidus External
**GPi**	Globus Pallidus Internal
**GU**	Gustatory Area
**IAD**	InfralimbicArea
**IAM**	Interanteromedial Nucleus
**IL**	Infralimbic Area
**LD**	Lateral Dorsal Nucleus
**MB**	Midbrain
**MD**	Mediodorsal Nucleus
**MRN**	Midbrain Reticular Nucleus
**MT**	Medial Terminal Nucleus
**ND**	Nucleus of Darkschewitsch
**NPC**	Nucleus of the Posterior Commissure
**MOp**	Primary Motor Area
**MOs**	Secondary Motor Area
**ORB**	Orbital Area
**PAG**	Periaqueductal Gray
**PAL**	Pallidum
**PCN**	Paracentral Nucleus
**PERI**	Perirhinal Area
**PF**	Parafascicular Nucleus

**Abbreviation**	
**PL**	Prelimbic Area
**PPN**	Pedunculopontine Nucleus
**PT**	Parataenial Nucleus
**RH**	Rhomboid Nucleus
**RN**	Red Nucleus
RPF	Retroparafascicular Nucleus
**RT**	Reticular Nucleus
**RSP**	Restrosplenial Area
**SMT**	Submedial Nucleus
**SNc**	Substantia Nigra Pars Compacta
**SNr**	Substantia Nigra Pars Reticulata
**SSp**	Primary Somatosensory Area
SSp-bfd	Barrelfield Area
SSp-ll	Lower Limb Area
SSp-m	Mouth Area
SSp-n	Nose Area
SSp-tr	Trunk Area
SSp-ul	Upper Limb Area
SSp-un	Undefined Area
**SSs**	Secondary Somatosensory Area
**TEa**	Temporal Association Area
**TH**	Thalamus
**VAL**	Ventral Anterior-lateral Complex
**VISC**	Visceral Area
**VISp**	Primary Visual Area
VISa	Anterior Visual Area
VISal	Anterolateral Visual Area
VISam	Anteromedial Visual Area
VISpm	Posteromedial Visual Area
VISrl	Rostrolateral Visual Area
VISl	Lateral Area
**VM**	Ventral Medial Nucleus
**VPL**	Ventral Posterolateral Nucleus
**VPM**	Ventral Posteromedial Nucleus
**VTA**	Ventral Tegmental Area
